# Biochemical Components Associated With Microbial Community Shift During the Pile-Fermentation of Primary Dark Tea

**DOI:** 10.3389/fmicb.2018.01509

**Published:** 2018-07-10

**Authors:** Qin Li, Shuo Chai, Yongdi Li, Jianan Huang, Yu Luo, Lizheng Xiao, Zhonghua Liu

**Affiliations:** ^1^Key Laboratory of Tea Science of Ministry of Education, Hunan Agricultural University, Changsha, China; ^2^Hunan Provincial Key Laboratory of Crop Germplasm Innovation and Utilization, Hunan Agricultural University, Changsha, China; ^3^National Research Center of Engineering Technology for Utilization of Functional Ingredients from Botanicals, Hunan Agricultural University, Changsha, China; ^4^Collaborative Innovation Centre of Utilization of Functional Ingredients from Botanicals, Hunan Agricultural University, Changsha, China; ^5^Institute of Soil and Water Resources and Environmental Sciences, Zhejiang University, Hangzhou, China

**Keywords:** primary dark tea, food fermentation, microbial community, biochemical components, Illumina MiSeq sequencing

## Abstract

Primary dark tea is used as raw material for compressed dark tea, such as Fu brick tea, Hei brick tea, Hua brick tea, and Qianliang tea. Pile-fermentation is the key process for the formation of the characteristic properties of primary dark tea, during which the microorganism plays an important role. In this study, the changes of major chemical compounds, enzyme activities, microbial diversity, and their correlations were explored during the pile-fermentation process. Our chemical and enzymatic analysis showed that the contents of the major compounds were decreased, while the activities of polyphenol oxidase, cellulase, and pectinase were increased during this process, except peroxidase activity that could not be generated from microbial communities in primary dark tea. The genera *Cyberlindnera*, *Aspergillus*, *Uwebraunia*, and *Unclassified Pleosporales* of fungus and *Klebsiella*, *Lactobacillus* of bacteria were predominant in the early stage of the process, but only *Cyberlindnera* and *Klebsiella* were still dominated in the late stage and maintained a relatively constant until the end of the process. The amino acid was identified as the important abiotic factor in shaping the microbial community structure of primary dark tea ecosystem. Network analysis revealed that the microbial taxa were grouped into five modules and seven keystone taxa were identified. Most of the dominant genera were mainly distributed into module III, which indicated that this module was important for the pile-fermentation process of primary dark tea. In addition, bidirectional orthogonal partial least squares (O2PLS) analysis revealed that the fungi made more contributions to the formation of the characteristic properties of primary dark tea than bacteria during the pile-fermentation process. Furthermore, 10 microbial genera including *Cyberlindnera*, *Aspergillus*, *Eurotium*, *Uwebraunia, Debaryomyces*, *Lophiostoma*, *Peltaster*, *Klebsiella*, *Aurantimonas*, and *Methylobacterium* were identified as core functional genera for the pile-fermentation of primary dark tea. This study provides useful information for improving our understanding on the formation mechanism of the characteristic properties of primary dark tea during the pile-fermentation process.

## Introduction

Dark tea is a unique kind of microbial fermented teas, which is native to many regions of China, including Hunan, Yunnan, and Hubei provinces (Zhang et al., 2013). The manufacturing process of dark tea can be divided into primary processing stage and reprocessing stage. During the primary processing stage, primary dark tea was produced and was mainly used as material for compressed dark tea, including Fu brick tea, Hei brick tea, Hua brick tea, and Qiangliang tea. The manufacturing process of primary dark tea covers the following steps, harvesting fresh tea leaves, fixing, rolling, pile-fermentation, and drying processes (Li et al., 2017). In the above processes, the pile-fermentation, a kind of solid-state fermentation, is the key process in the production of primary dark tea. The pile-fermentation process of primary dark tea is characterized by the growth and succession of microbial communities, and the microorganism plays a key role in the formation of the characteristic properties of primary dark tea during the pile-fermentation process (Wang et al., 1991a).

A comprehensive study of microbial community succession could help to understand that how the sensory quality of primary dark tea was formed during the pile-fermentation process. Some kinds of microbial genera, including *Candida* sp., *Aspergillus niger*, and *Penicillium* in fungal community and *Non-spore bacteria*, *Bacillus*, and *Coccus* in bacterial community, were identified as the dominant microbial genera by the culture-dependent method during the pile-fermentation process of primary dark tea (Wen and Liu, 1991). However, the culture-dependent method is of limited value for identifying the microbial communities due to the limitation of cultural media (Li et al., 2011). Hence, the culture-independent methods, including terminal restriction fragment length polymorphism (T-RFLP), denaturing gradient gel electrophoresis (DGGE), and high-throughput sequencing technology (i.e., Illumina MiSeq sequencing) that use direct analysis of the diversity of ribosomal RNA genes with no culture step (Amann and Ludwig, 2000), were introduced for identifying the structure of microbial communities in compressed dark tea, such as Fu brick tea and Pu-erh tea (Abe et al., 2008; Xu et al., 2011; Zhao et al., 2013; Fu et al., 2016; Li et al., 2017). The genera *Aspergillus*, *Blastobotrys*, *Bacillus*, and *Enterobacteriaceae* were the major microbial communities in Pu-erh tea (Abe et al., 2008; Zhao et al., 2013), and *Aspergillus*, *Cyberlindnera*, *Candida*, *Penicillium*, *Eurotium*, *Beauveria*, *Debaryomyces*, *Pestalotiopsis*, *Pichia*, *Rhizomucor*, and *Verticillium* were the dominant microbial communities in Fu brick tea by PCR-DGGE (Xu et al., 2011) and high-throughput sequencing technology (Li et al., 2017).

During the pile-fermentation process, some new transformation products were generated in tea leaves. Our previous study showed that the power of substance transformation in tea leaves were mainly derived from the microbial enzymes, which were secreted from the microorganism during the pile-fermentation process of primary dark tea (Wang et al., 1991a). These transformations resulted in the formation of the characteristic properties of primary dark tea. The microbial metabolism, which produce the secreted enzymes such as polyphenol oxidase (PPO), cellulase (CEL), and pectinase (PEC), thereby providing the effective biochemical power to oxidation of catechins, decomposition of cellulose, and hydrolyses of pectin and proteins. The microbial enzymatic action played an important role in the formation of the characteristics of primary dark tea (Liu et al., 1991). During the pile-fermentation process, the total contents of polyphenol, catechin, and soluble carbohydrate were decreased, and the catechin composition varied irregularly (Wang et al., 1991c). The content of total amino acid (AA) was decreased, especially theanine and glutamic acid were decreased significantly; by contrast, the content of indispensable AAs, such as lusine, phenylalanine, leucine, isoleucine, methionine, and valine, were increased remarkably. And the levels of caffeine, theobromine, and theophylline were not significantly variated (Wang et al., 1991b).

The succession of microbial community in primary dark tea during pile-fermentation process results in a dynamic taste and aroma of primary dark tea. Variation of major chemical compounds and enzyme activities in fermented tea has been studied (Liu et al., 1991; Wang et al., 1991b,c). The culturable microorganisms have also been identified in primary dark tea during the pile-fermentation process (Wen and Liu, 1991). However, the associations among microbiota and chemical compounds or enzyme activities, and so far the core functional microbiota were not clarified in primary dark tea. Thus, the assembly and dynamics of microbial community were characterized by Illumina MiSeq sequencing during the pile-fermentation of primary dark tea. The changes of major chemical compounds content and enzyme activity were also investigated during this process. Based on these results, the relationship between microbiota and chemical compounds or enzyme activities was investigated by bidirectional orthogonal partial least squares (O2PLS). Finally, the core functional microorganisms were identified by comparison of the comprehensive importance of microorganisms correlated with the formation of characteristic properties of primary dark tea. The knowledge provide a new insight for better understanding on the mechanism of pile-fermentation in primary dark tea.

## Materials and Methods

### Tea Leaf Samples and Process Characterization

Sampling of primary dark tea was carried out in Ba Jiao tea factory (Yiyang, Hunan province, China). The manufacturing process of primary dark tea was performed as described by DB43/T660-2011 with a little modification (Liu et al., 2011). Briefly, mature tea leaves (one bud and three to six leaves) were plucked from medium and sprinkled with water then fixing at 280–320°C. The fixing leaves rolled twice by machine. Then, the rolling leaves (about 930 kg) were piled 1.1 m high, with ambient temperature about 28°C and moisture content about 78% in pile-fermentation apparatus for 12 h. Auto drying machine was used for drying the fermentation leaves at 120–130°C twice or drying in the sun. Fifteen samples were collected from three independent batches of primary dark tea at different time of pile-fermentation process: pile-fermentation 0-, 3-, 6-, 9-, and 12-h stages. The samples were packaged in sterile polyethylene bags, transported to the laboratory, and stored at −80°C until required.

### Chemical Compounds and Enzyme Activity Analysis

The content of water extract (WE) was determined according to the National Standard GB/T 8305-2013 (Tea-Determination of water extracts content) (Zhou et al., 2013). Total polyphenols (TP) and flavonoids (FLA) content were determined by a colorimetric method, as described previously (Li et al., 2013). Free AA content was determined according to the National Standard GB/T 8314-2013 (Tea-Determination of free amino acids content) (Xu et al., 2013). Soluble sugar (SS) content was determined according to sulfuric acid–anthrone colorimetric method, as described previously (DuBois et al., 1956). All analyses were performed three times.

Extraction of enzyme extracts was based on our previous method with slight modifications (Teng et al., 2017). 1.25-g tea leaves were ground with 0.75-g polyvinylpolypyrrolidone (PVPP) in a mortar on ice. The crude enzyme was extracted with 25-mL citrate buffer (pH 5.6) on ice for 12 h. Subsequently, the mixture was centrifuged at 15,000 × *g* for 30 min at 10°C, and the clear supernatant was collected and filled to 25 mL with citrate buffer. The clear supernatant was used in all enzyme assays. The activities of PPO, peroxidase (POD), CEL, and PEC were determined according to previous studies (Wood and Bhat, 1988; Huang, 1997; Biz et al., 2014). All analyses was performed three times. The detail approaches were described in Supplementary Material 1.

### DNA Extraction and Sequencing

Each tea sample (5 g) was mixed with sterile water (25 mL), stirred thoroughly, filtered through three layers of coarse sterile gauze to remove large particles, and centrifuged at 10,000 × *g* for 10 min at 4°C. The pellets were used for genomic DNA extraction. DNA from the sample was extracted using PowerSoil^®^ DNA Isolation Kit (Mo Bio Laboratories Inc., Carlsbad, CA, United States), according to the manufacturer’s protocols. The duplicate samples were pooled together for one high-throughput sequencing. The V4 region of 16S was amplified using the primer pair 515F (5′-GTGCCAGCMGCCGCGG-3′) and 806R (5′-GGACTACHVGGGTWTCTAAT-3′) (Xiong et al., 2012; Fan et al., 2014); the ITS rRNA gene was amplified using the primer pair ITS1F (5′-CTTGGTCATTTAGAGGAAGTAA-3′) and ITS2 (5′-GCTGCGTTCTTCATCGATGC-3′) (Mukherjee et al., 2014). The forward and reverse primers were tagged with adapter, pad, and linker sequences. The reaction mixture (50 μL) contained 25 μL of Taq PCR Master Mix (QIAGEN), 0.2 μM of each primer, and 10 ng DNA templates. Thermal cycling consisted of initial denaturation at 94°C for 4 min, followed by 35 cycles of denaturation at 94°C for 40 s, annealing at 54°C (ITS) and 52°C (16S) for 45 s, elongation at 72°C for 50 s, and finally elongation at 72°C for 10 min. Negative control was treated similarly with the exclusion of template DNA and failed to produce visible PCR products. For each sample, three independent reactions were performed. PCR products were mixed in equimolar ratios and mixture PCR products were purified with GeneJET Gel Next^®^ Ultra^TM^ DNA Library Prep Kit for Illumina (NEB, United States) following manufacturer’s recommendations. The library quality was assessed on the Qubit^@^2.0 Fluorometer (Thermo Scientific) and Agilent Bioanalyzer 2100 system. Finally, the library was sequenced on an Illumina MiSeq platform using paired-end sequencing by Novogene Inc. (Beijing, China).

### Data Processing and Statistical Analysis

Raw reads files were demultiplexed, quality filtered, and analyzed using the Quantitative Insights Into Microbial Ecology (QIIME, v1.7.0) pipeline (Caporaso et al., 2010). In brief, reads with less than length 220 bp and ambiguous bases were discarded. Chimeras were removed using UCHIME as implemented in QIIME. The effective tags were binned into operational taxonomic units (OTUs) by UCLUST based on 97% pairwise identity (Edgar et al., 2011). The most abundant sequence from each OTU was selected to represent the OUT. OTUs were classified taxonomically using a QIIME-based wrapper of the Ribosomal Database Project (RDP) classifier against UNITE (User-friendly Nordic ITS Ectomycorrhiza Database) and INSD (International Nucleotide Sequence Databank) database (Caporaso et al., 2010). All identified OTU sequences assigned to chloroplast and mitochondrion origins were removed from the dataset.

The alpha diversity (Shannon index), richness (Chao1 index), Good’s coverage, and rarefaction curve were performed by Mothur (Schloss et al., 2011). In addition, rank abundance curves, principal components analysis (PCA) and non-metric multidimensional scaling (NMDS) were generated by using R package vegan (version 3.4.0^1^). CANOCO software (version 4.5^2^) was used to conduct multivariate analysis using detrended correspondence analysis (DCA) and redundancy analysis (RDA). One-way ANOVA test, Pearson’s correlation coefficients, and *P*-values were calculated with IBM SPSS 19.0.^3^ All results were presented as the mean value ± SD. Differences between groups were declared significant at *P* < 0.05.

### Co-occurrence Network Analysis and O2PLS Analysis

The co-occurrence patterns of microbial community in the pile-fermentation process of primary dark tea were constructed and analyzed using the online Molecular Ecological Network Analyses Pipeline (MENAP^4^; Deng et al., 2012). The OTUs, which presented in at least six samples, were used for pairwise Pearson correlation calculation, for which a proper threshold was identified based on the random matrix theory (RMT) approach. Network topological properties were also calculated using MENAP. O2PLS analysis was performed using the SIMCA-P 14 (Version 14.1.0.2047), according to the previous study (Wang et al., 2016). The X-matrix was designed as the microbiota dataset, and the Y-matrix was designed as the chemical compounds and enzyme activities datasets. Finally, the networks and the correlation among microbiota, chemical compounds, and enzyme activities were visualized using the Cytoscape software (Version 3.5.1).

## Results

### Major Chemical Compounds Along With the Time Gradient in the Pile-Fermentation of Primary Dark Tea

**FIGURE 1:**
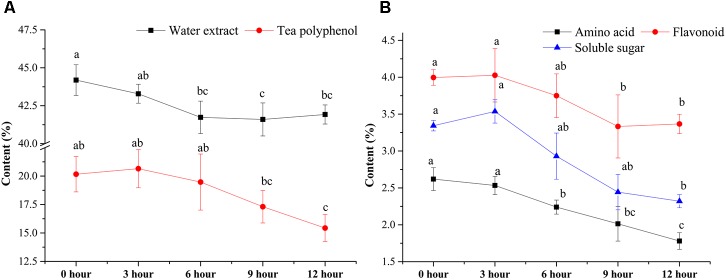
Changes in the major chemical compounds during the pile-fermentation process of primary dark tea. Water extract and tea polyphenol **(A)**, amino acid, flavonoid, and soluble sugar **(B)**.

The contents of major chemical compounds, including WE, TP, FLA, AA, and SS, were analyzed during the pile-fermentation process of primary dark tea. As shown in **Figure 1**, all the contents of major compounds were significantly decreased after the pile-fermentation process (*P* < 0.05). The concentrations of WE and AA were significantly decreased beginning at 6-h stage, 5.43 and 47.90% decrease was observed at 12-h stage compared with 0-h stage, respectively. The levels of TP and FLA were slightly decreased from 0-h stage to 6-h stage and sharply decreased at 9-h stage until the end of process, where 31.68 and 18.84% decrease was observed. A slightly increased content of SS was found from 0-h stage to 3-h stage, but a 44.24% decrease of SS was also observed at the end of process (**Figure 1**).

### Change of Enzyme Activity During the Pile-Fermentation of Primary Dark Tea

**FIGURE 2:**
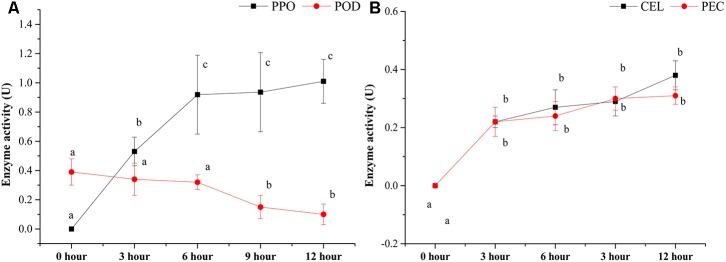
Changes in the enzyme activities during the pile-fermentation process of primary dark tea. PPO, polyphenol oxidase; POD, peroxidase **(A)**; CEL, cellulase; PEC, pectinase **(B)**.

Several enzyme activities, including PPO, POD, CEL, and PEC, were also detected during the pile-fermentation of primary dark tea (**Figure 2**). The activities of PPO, CEL, and PEC were significantly increased after the fermentation process, but the POD activity was significantly decreased in this process. The PPO activity was significantly increased from 0-h stage (not detected) to 0.92 ± 0.27 U at 6-h stage and remained stable until the end of process. And the levels of CEL and PEC activities were markedly increased from 0-h stage (not detected) to 0.22 ± 0.03 and 0.21 ± 0.05 U at 3-h stage then slightly increased until the end of process. But the POD activity was significantly decreased by 74.56% after pile-fermentation process.

### Microbial Community Diversity During the Pile-Fermentation Process

**FIGURE 3:**
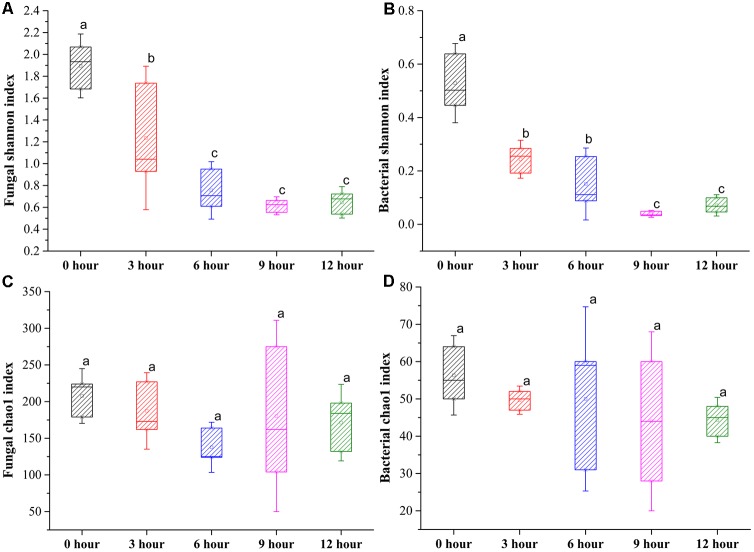
Changes in Shannon and Chao1 index of fungal **(A,C)** and bacterial **(B,D)** community during the pile-fermentation process of primary dark tea.

In total, 1,401,447 valid fungal sequences and 418,889 valid bacterial sequences across all samples clustered into 297 fungal OTUs and 77 bacterial OTUs after trimming and filtration at 97% sequence identify. The values of Good’s coverage were >99% for all sequences from five groups (**Supplementary Table S1**). The rarefaction curves for all samples almost reached the saturation phase, suggesting that few new microorganisms would possibly be identified by increasing the sequencing depth, and the majority of microorganisms for the samples had already been captured in the current analysis (**Supplementary Figure S1**). The Shannon index indicated that the diversity of fungus and bacteria was significantly decreased from 0-h stage to 6-h stage and maintained relatively constant until the end of the process. The Chao1 index indicated that the richness of fungus and bacteria was slightly decreased during the pile-fermentation process, but no significant difference was observed at the end of the process (**Figure 3**). In addition, the rank abundance curves of fungus and bacteria also showed that a slightly decreased richness and evenness of fungus and bacteria after the pile-fermentation process of primary dark tea (**Supplementary Figure S2**).

**FIGURE 4:**
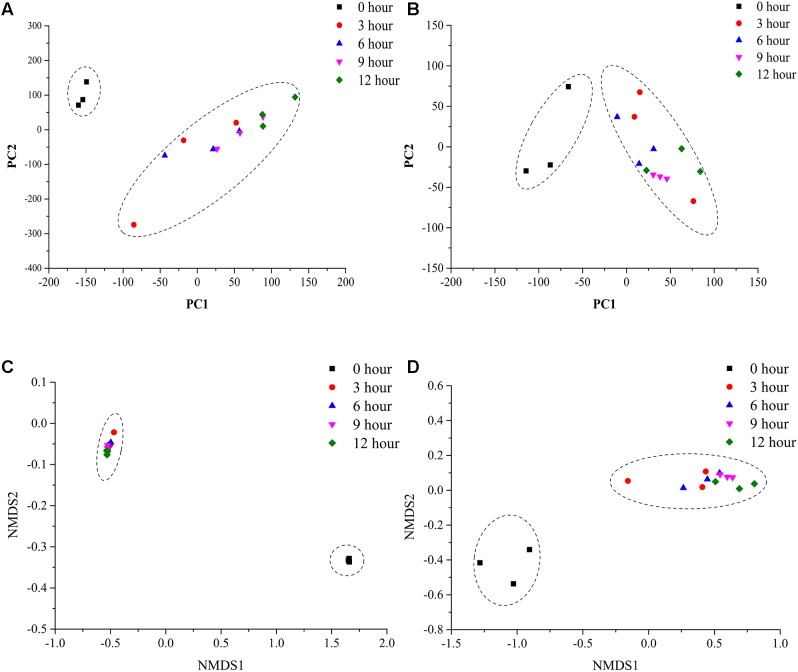
PCA and NMDS analysis of microbial communities in sample during the pile-fermentation process of primary dark tea. Fungal PCA **(A)**, bacterial PCA **(B)**, fungal NMDS **(C)**, and bacterial NMDS **(D)**.

To assess the microbial β-diversity, the PCA and NMDS analyses were employed to investigate the overall difference among bacterial and fungal community structure during the pile-fermentation process of primary dark tea. The results indicated that all samples could be separated into two groups with based on Euclidean distance and Bray–Curtis distance, including group I (0-h stage) and group II (3-h stage-12-h stage; **Figure 4**).

### Dynamic Shift of Microbial Community During the Pile-Fermentation Process of Primary Dark Tea

**FIGURE 5:**
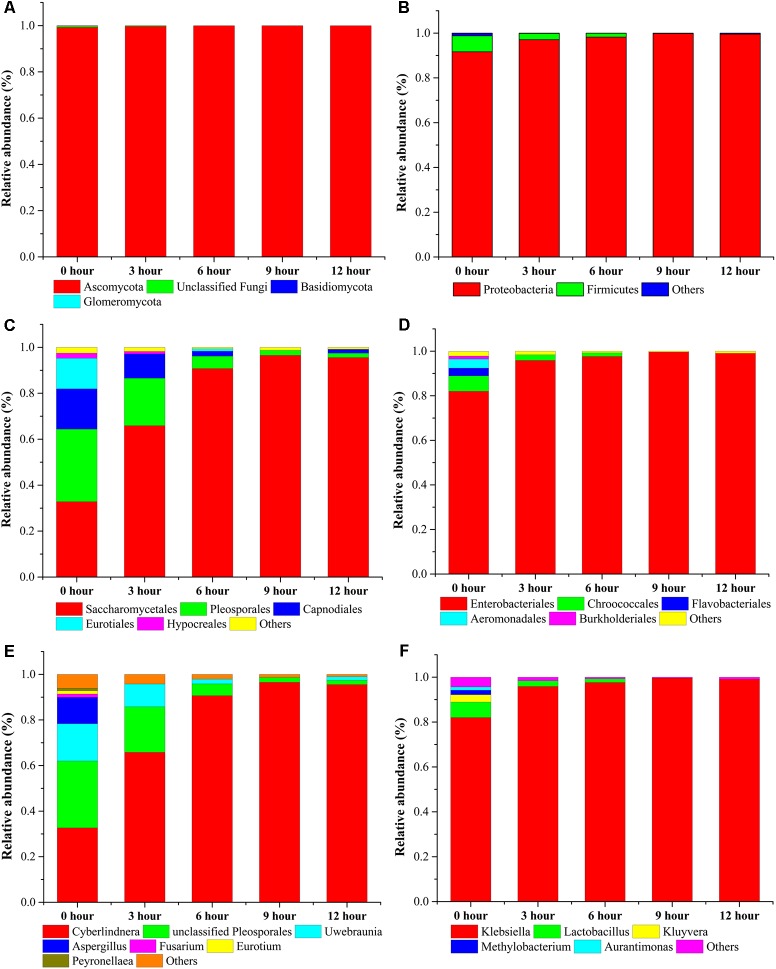
Microbial taxonomic compositions showing the microbial successions at phylum: **(A)** fungus, **(B)** bacteria; order: **(C)** fungus, **(D)** bacteria; and genus: **(E)** fungus, **(F)** bacteria level during pile-fermentation process of primary dark tea. The taxonomic abundance <1% were classified into “others.”

For microbial diversity and succession, 4 phyla, 53 classes, 47 orders, 216 families, and 153 genera in fungal community and 5 phyla, 9 classes, 20 orders, 35 families, and 55 genera in bacterial community were identified during the pile-fermentation process of primary dark tea. At the genus level of fungal composition, the four most dominant fungal genera, including *Cyberlindnera*, *Uwebraunia*, *Aspergillus*, and *Unclassified Pleosporales* constituted 89.91–99.47% of all sequences. *Cyberlindnera* dominated the whole fermentation process, with the relative abundance dramatically increased from the initial stage (0-h stage) 32.63–95.52% in 12-h stage. However, *Uwebraunia* dramatically decreased from 16.37% in 0-h stage to 1.64% in 12-h stage. *Aspergillus* and *Unclassified Pleosporales* accounted for 11.57 and 29.35% of all sequences in 0-h stage and was significantly decreased to 0.33% in 3-h stage and 5.18% in 6-h stage, then remained relative stable until the end of the pile-fermentation process. Changes in some small proportions in the fungal groups *Fusarium* (1.45–0.45%), *Eurotium* (1.44–0.31%), and *Peyronellaea* (1.11–0.23%) were also observed during the pile-fermentation process (**Figures 5A,C,E**). For the bacterial composition, the dominant genera *Klebsiella* and *Lactobacillus* accounted for 88.87–99.48% during the pile-fermentation process of primary dark tea. *Klebsiella* dominated the whole fermentation process, with the relative abundance gradually increased from the initial stage (0-h stage) 81.99–99.06% in 12-h stage. However, *Lactobacillus* dramatically decreased from 6.88% in 0-h stage to 0.41% in 12-h stage. Variations in some small proportions in the bacterial groups *Kluyvera* (3.31–0.29%), *Methylobacterium* (2.02–0.04%), and *Aurantimonas* (1.44–0.02%) were also observed during the pile-fermentation process of primary dark tea (**Figures 5B,D,F**).

### Interaction Network Between Microbial Communities

**FIGURE 6:**
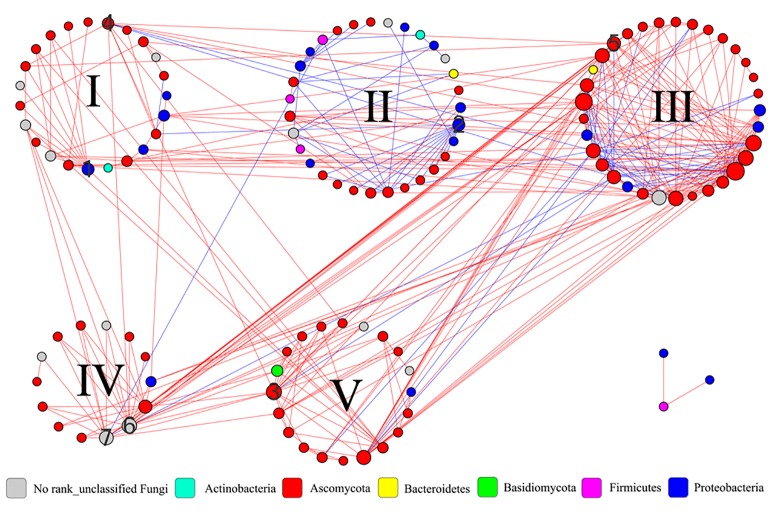
Interaction networks in the microbial communities of primary dark tea. The nodes of network were group into five modules. The co-occurring networks are colored by phylum. The size of each node is proportion to the number of connections (that is, degree), and the red edge indicates a positive interaction and the blue edge indicated a negative interaction between two nodes. The numbers are indicated as follows: 1∼3 the module hubs bOTU8, bOTU21, and fOTU7. 4∼7 the connectors fOTU21, fOTU30, fOTU58, and fOTU162. I∼V indicated the number of modules.

To understand the interactions and connectivity within microbial communities during the pile-fermentation process of primary dark tea, we identified molecular ecological networks (MENs) using a novel RMT-based approach (Deng et al., 2012). In RMT analysis, the similarity threshold was determined to be 0.79. A total of 339 pairs of significant and robust correlations (edges) were identified from 124 nodes (28 nodes for bacteria and 96 nodes for fungus) (**Figure 6**). Specifically, 267 pairs of positive correlations and 72 pairs of negative correlations were identified in the network. Moreover, the value of modularity index for network was 0.446 (>0.4), suggesting the network has a modular structure (Newman, 2006) and the network global properties were showed in **Supplementary Table S2**.

All nodes in the network were assigned to seven phyla, including four bacterial phyla (*Actinobacteria*, *Bacteroidetes*, *Firmicutes*, and *Proteobacteria*) and three fungal phyla (*Ascomycota*, *Basidiomycota*, and *Unclassified Fungi*); the individual node’s properties were shown in **Supplementary Table S3**. All nodes could be grouped into five modules and the module compositions were shown in **Supplementary Figure S3** at the genus level. The majority of nodes (117, 94.35%) were peripherals with most of their links inside their own modules during the pile-fermentation process of primary dark tea. A total of four nodes were identified as connectors, which were derived from *Staninwardia* (phyla *Ascomycota*), *Saccharata* (phyla *Ascomycota*), and two *Unclassified Fungi* nodes. Three nodes were identified as module hubs, which were derived from *Aeromonas* (phyla *Proteobacteria*), *Acidovorax* (phyla *Proteobacteria*), and *Unclassified Pleosporales* (phyla *Ascomycota*) (**Supplementary Figure S4**). These keystone species served as gatekeepers in the ecological functions of the microbial community, implying that they may play critical roles in maintaining the stability of structure and function of ecological communities during the pile-fermentation of primary dark tea.

### Relationship Among Microbiota, Chemical Compounds, and Enzyme Activities

**FIGURE 7:**
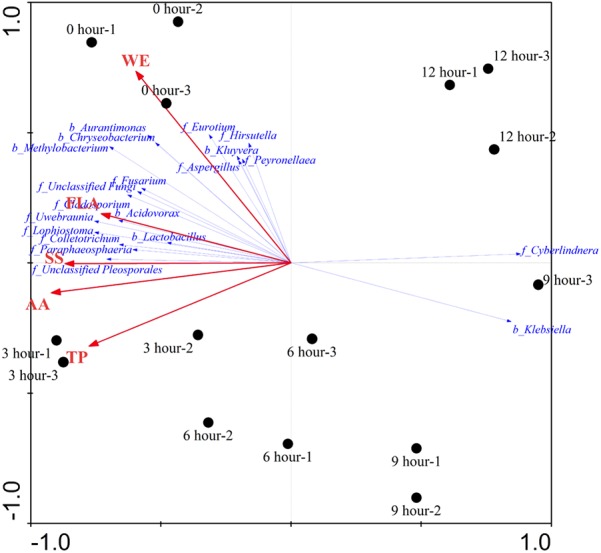
Redundancy analysis (RDA) showing the relationship between microbial structure and major chemical compounds concentrations during the pile-fermentation process of primary dark tea. The red arrow indicated the compounds variances; the blue arrow indicated the important genera of microbial communities (relative abundance >0.01%). Filled circles indicated the samples during the pile-fermentation of primary dark tea. WE, water extract; TP, tea polyphenol; AA, amino acid; FLA, flavonoid; SS, soluble sugar.

The effects of major chemical compounds on the structure of microbial community during the pile-fermentation process of primary dark tea were evaluated by the DCA and RDA. The result of DCA indicated that the gradient length of the axis was less than 3 SD (lengths of gradient 1.543, 0.409, 0.353, 0.861). Therefore, the linear model of RDA was considered to be the appropriate ordination method for direct gradient analysis. As shown in **Figure 7**, the pattern of microbial communities exhibited a continuous succession along with the time gradient during the pile-fermentation process. A sharply change from 0-h stage to 3-h stage and a gradual variation occurred in from 3-h stage to 9-h stage, then a greater variation occurred in the transitions from 9-h stage to 12-h stage. RDA revealed that there was a significant relationship between the structure of microbial community and major chemical component factors (*P* = 0.014). All five chemical component factors (i.e., WE, TP, AA, FLA, and SS) explained 74.5% (*P* < 0.05) of the total variability in the microbial community. The results of Monte Carlo permutation tests showed that the variability of the microbial composition was strongly related to AA (*P* = 0.026). The first ordination RDA axis (axis 1), which was strongly related with AA and SS, explained 72.6% of the variability in the microbial composition. The second ordination RDA axis (axis 2) was mainly related to WE and explained 1.1% of the total variability in the microbial community.

**FIGURE 8:**
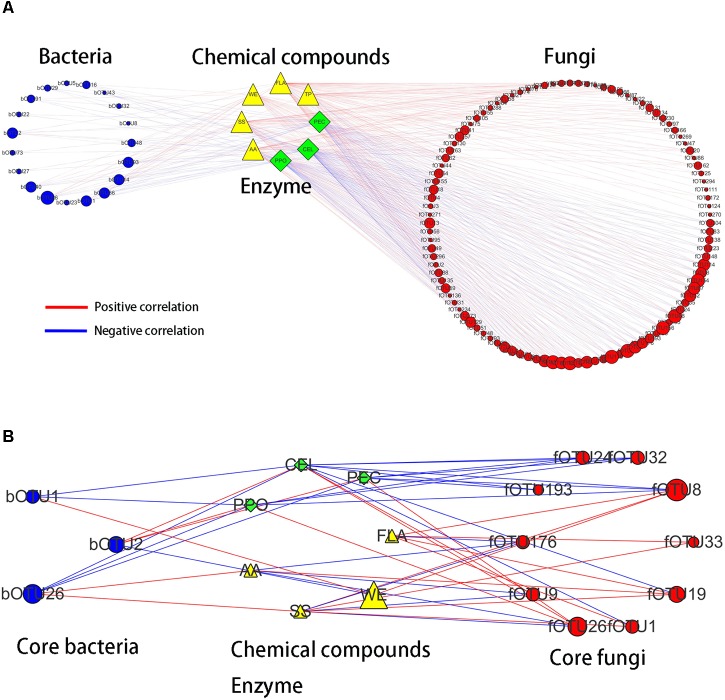
Correlation analyses among microbiota, chemical compounds, enzyme activities **(A)** and the core microorganisms **(B)** in primary dark tea during pile-fermentation process.

Bidirectional orthogonal partial least squares method was used to analyze the associations among microbiota, chemical compounds, and enzyme activities during the pile-fermentation process of primary dark tea. It was shown *R*_2_ and *Q*_2_ of the model was 0.938 and 0.518, respectively, thereby suggesting that O2PLS method was well fitted for analysis and prediction. Three latent variables were identified, with the microbiota (X dataset)-predictive structures accounting for 80.8% of the total variation in the X dataset, the chemical compounds and enzyme activities (Y dataset)-predictive structures accounting for 99.9% of the total variation in the Y dataset. According to the values of predictive component variable importance in the projection (VIP_pred_) and the correlation coefficient between microbiota and chemical compounds or enzyme activities, 123 OTUs (VIP_pred_ > 1.0 and |*r*| > 0.6), including 19 bacterial OTUs and 104 fungal OTUs, had important effects on chemical compounds and enzyme activities (**Figure 8A**). In total, 106 OTUs including 13 bacterial OTUs and 93 fungal OTUs were correlated with chemical compounds. And 83 OTUs including 17 bacterial OTUs and 66 fungal OTUs were correlated with enzyme activities. These results suggested that the fungus were more important for primary dark tea production than bacteria.

In order to identify the core functional microorganisms in the pile-fermentation process of primary dark tea, several conditions should be considered: (a) detected stably in pile-fermentation process; (b) the VIP_pred_ value must be greater than 1; (c) the correlation coefficient must be greater than 0.7; (d) the number of microbe highly correlated with (|*r*| > 0.7) chemical compounds and enzyme activity was greater than 1. Based on these, 13 OTUs including *Klebsiella* (bOTU2), *Aurantimonas* (bOTU1), and *Methylobacterium* (bOTU26) of bacterial genera and *Cyberlindnera* (fOTU176, fOTU26, and fOTU9), *Aspergillus* (fOTU24), *Uwebraunia* (fOTU1 and fOTU8), *Debaryomyces* (fOTU33), *Eurotium* (fOTU193), *Lophiostoma* (fOTU19), and *Peltaster* (fOTU32) of fungal genera were identified as core functional microorganisms for the pile-fermentation process of primary dark tea. As shown in **Figure 8B**, *Aurantimonas* was mainly responsible for the changes of CEL, WE, and PPO. *Klebsiella* was mainly responsible for WE, PPO, PEC, and CEL. *Methylobacterium* was mainly responsible for AA, SS, PPO, PEC, and CEL. *Cyberlindnera* was mainly responsible for AA, SS, FLA, PPO, PEC, and CEL. *Uwebraunia* was mainly responsible for WE, FLA, SS, PPO, PEC, and CEL. *Peltaster* was mainly responsible for PPO, PEC, and CEL. *Lophiostoma* was mainly responsible for AA, SS, FLA, and CEL. *Eurotium* was mainly responsible for PEC and CEL. *Aspergillus* was mainly responsible for PPO, PEC, and CEL.

## Discussion

Diverse and rich communities of bacteria and fungi with potential health benefits have been identified from various fermented food and beverages (Bourdichon et al., 2012). Microbiota inhabiting in tea leaves is of great importance for the distinctive biochemical characteristics of primary dark tea. To the best of our knowledge, the present study is the first work to extensively investigate the structural differentiation of microbial communities by Illumina MiSeq sequencing and linked with the major chemical compounds or enzyme activity during pile-fermentation process of primary dark tea.

Our chemical and enzymatic analysis showed that all major chemical compounds content were decreased, while the activities of PPO, CEL, and PEC were increased during the pile-fermentation process of primary dark tea, except POD. Previous studies revealed that the decrease of chemical compounds mainly resulted from enzymatic reaction of secretive enzyme of microbial communities during the fermentation process (Wang et al., 1991a). The activities of PPO, CEL, and PEC sourced from tea leave were inactivated by high temperature in fixing process. Very low activity of POD was remained after the fixing process, which is a kind of hyper-thermoresistant enzyme and could not be secreted by microbial communities during the pile-fermentation of primary dark tea (Liu et al., 1991). The activities of PPO, CEL, and PEC were increased along with the growth of microbial communities and provided the effective biochemical power to the biotransformation of chemical compounds in the tea leaves during the pile-fermentation process. The catechin was oxidation by PPO, which resulted in the decrease of TP content during the process. The decrease of SS maybe resulted from the growth of microorganisms, which was used as the most important carbon source for microbial communities (Wang et al., 1991c). The decreased of AA was also used as the mainly nitrogen source for the growth of microorganisms (Wang et al., 1991b). The genera *Cyberlindnera*, *Aspergillus*, *Uwebraunia*, and *Unclassified Pleosporales* of fungus and *Klebsiella* and *Lactobacillus* of bacteria were predominant at 0-h stage of process, but after 3-h stage and 6-h stage, only *Klebsiella* and *Cyberlindnera* were still dominated in fungal and bacterial community, and maintained a relatively constant until to the end of pile-fermentation process. This shift pattern of microbial communities might be because of the environment changes in the tea pile during fermentation process. Our RDA result also revealed that AA was the important factor shaping the microbial structure. AA, caffeine, theobromine, and theophylline were the major nitrogenous compounds of primary dark tea, but caffeine, theobromine, and theophylline were heterocyclic compounds, which could hardly be used by microorganisms (Wang et al., 1991b). Therefore, AA is the most important nitrogen source of microorganisms during the pile-fermentation process of primary dark tea. Co-occurrence analysis showed that the OTUs were grouped into five modules, which might perform different functions during the pile-fermentation process of primary dark tea. In this study, most of the dominant genera were distributed in module III (**Supplementary Figure S3**), such as *Cyberlindnera*, *Aspergillus*, and *Uwebraunia* of fungal communities and *Klebsiella* of bacterial community, which might be mainly related to carbon and nitrogen metabolism during pile-fermentation process (Bekatorou et al., 2006; Du et al., 2006; Cho et al., 2012).

Bidirectional orthogonal partial least squares approach was used to integrate the microbiota, chemical compounds, and enzyme activities dataset in order to dig into the association among them in primary dark tea during pile-fermentation process. The results suggested that fungus were more important for primary dark tea production than bacteria. In addition, *Cyberlindnera*, *Aspergillus*, *Debaryomyces*, *Uwebraunia, Eurotium*, *Lophiostoma*, and *Peltaster* of fungal genera and *Klebsiella*, *Aurantimonas*, and *Methylobacterium* of bacterial genera were identified as the core microorganisms during the pile-fermentation process of primary dark tea.

The fungal genus *Cyberlindnera* contains 23 recognized teleomorphic species and 12 *Candida* species (Chang et al., 2012), in which the *Cyberlindnera jadinii* was applicated in the production of papaya wine (Pinrou et al., 2010) and prevented the growth of spoilage yeasts in cheese (Liu and Tsao, 2009). This specie is a close relative of *Candida utilis* (also referred to as *Torula* yeast), which was cultivated on economical waste liquors and used in the food and feed industries as a nutritious, well-tasting, and safe source of single-cell protein. *Cyberlindnera* (*Williopsis*) *saturnus* was reported that had the ability for producing significant amounts of sweetener xylitol from xylose and corn cob hydrolysate (Kamat et al., 2013). Moreover, some species of *Cyberlindnera*, which had a high capability of tannin tolerance, were also isolated from Miang (a fermented food product prepared from tea leaves) (Kanpiengjai et al., 2016). *Aspergillus* is the most important economically fungal genus (Samson et al., 2014), and many species within this genus are used in biotechnology for the production of various metabolites in many food fermentations (Bourdichon et al., 2012), such as soy sauce (Wicklow et al., 2007), miso (Harayama and Yasuhira, 1994), grape pomace (Botella et al., 2007), millet spirits (Takaya et al., 2007), and katsuobushi (Manabe, 2001). In all these cases, *Aspergillus* species which produce and secrete a variety of enzymes including α-amylases, glucoamylases, CELs, PECs, xylanases and other hemicellulase, and proteases, play an important role in the improvement of taste by decomposing proteins and/or lipids and producing unique flavors (Ward et al., 2005). *Eurotium*, which adopted the newly established principle “one fungus, one name” (Norvell, 2011), species formerly included in the genus *Eurotium* are displayed with their *Aspergillus* name (Samson et al., 2014), such as the dominant fungi *Eurotium cristatum* in Fu brick tea, which is called *Aspergillus cristatum* now (Ge et al., 2016; Li et al., 2017). Several indole alkaloids and indole diketopiperazine alkaloid were identified from the culture extract of *Aspergillus cristatum* and these compounds were proved to possess brine shrimp lethality, antibacterial activity against *E. coli*, radical scavenging activity against DPPH radicals, and exhibited marginal attenuation of 3T3L1 pre-adipocytes (Du et al., 2012, 2017; Zou et al., 2014). And non-mycotoxigenic strains of *Aspergillus repens* and *Aspergillus rubrum* are used as starter cultures in the manufacture of the traditional fermented food *katsuobushi*, from bonito (*Katsuwonus pelamis*) (Manabe, 2001). *Debaryomyces* contains 19 species (Ainsworth, 2014), in which the species *Debaryomyces hansenii* (anamorph *Candida famata*) is a significant species in foods and play an important role for the food industry, as it is used for surface ripening of cheese and meat products, over-production of riboflavin (vitamin B2), bioconversion of xylose into the sweetener xylitol, and potential synthesis of arabinitol and pyruvic acid (Satyanarayana and Kunze, 2009). And its enzyme activities improve tea quality by producing the sweet substance xylitol, vitamins, and other organic acids in Fu brick tea (Xu et al., 2011). *Uwebraunia* is a kind of plant pathogenic fungi, which was a synonym of *Dissoconium* (Crous et al., 2006), but the genus have not been well described, and the understanding of its roles in pile-fermentation systems of primary dark tea is limited.

The bacterial genus *Klebsiella* have shown an unbeatable production performance of 2,3-butanediol by fermentation from a wide range of substrates, including pentoses (xylose and arabinose), hexoses (glucose, mannose, and galactose), and disaccharides (sucrose, lactose, cellobiose) (Du et al., 2006; Cho et al., 2012). *Klebsiella* was also found in the fermentations of Pu-erh tea, soy sauce, and other fermented foods (Puerari et al., 2015; Zhao et al., 2015; Qin et al., 2016; Yang et al., 2017).

## Conclusion

In conclusion, a complex microbial community, inhabiting the primary dark tea ecosystem, plays important roles in the formation of quality of primary dark tea during the pile-fermentation process. The dynamic changes of major chemical compounds, enzyme activities, and microbial community structure, as well as the relationships among them, were clarified during the pile-fermentation process of primary dark tea. The results indicated that the fungi made more contributions to the formation of the characteristic properties of primary dark tea than bacteria during the pile-fermentation process. And 10 core functional microbial genera were identified in primary dark tea. These findings improved our understanding on the formation mechanism of the characteristic properties of primary dark tea during the pile-fermentation process.

## Author Contributions

QL, LX, ZL, and JH designed the study. QL conducted the experiment, analyzed the data, and drafted the manuscript. SC and YOL prepared the sample and conducted the chemical and enzyme analysis. YUL helped to revise the manuscript.

## Conflict of Interest Statement

The authors declare that the research was conducted in the absence of any commercial or financial relationships that could be construed as a potential conflict of interest.

## Footnotes

1 https://mirrors.tuna.tsinghua.edu.cn/CRAN/

2 http://www.canoco5.com/index.php/resources

3 https://www.ibm.com/analytics/us/en/technology/spss/

4 http://129.15.40.240/mena/

## Supplementary Material

FIGURE S1 Rarefaction curves of fungal OTUs **(A)** and bacterial OTUs **(B)** derived from primary dark tea.

FIGURE S2 Rank abundance curve of fungal OTUs **(A)** and bacterial OTUs **(B)** derived from primary dark tea.

FIGURE S3 Microbial community composition of each co-occurrence network module.

FIGURE S4 ZP plot showing distribution of OTUs based on their module-based topological roles.

TABLE S1 Comparison of microbial diversity estimation and coverage at 97% similarity from the sequencing analysis.

TABLE S2 Topological properties of network during the pile-fermentation process of primary dark tea.

TABLE S3 Individual node’s properties of network during the pile-fermentation process of primary dark tea.
