# Supplementary figures and images for: Biochemical Components Associated With Microbial Community Shift During the Pile-Fermentation of Primary Dark Tea
Source: Front Microbiol. 2018 Jul 10;9:1509. doi: 10.3389/fmicb.2018.01509 (PMC6048958; doi:10.3389/fmicb.2018.01509)

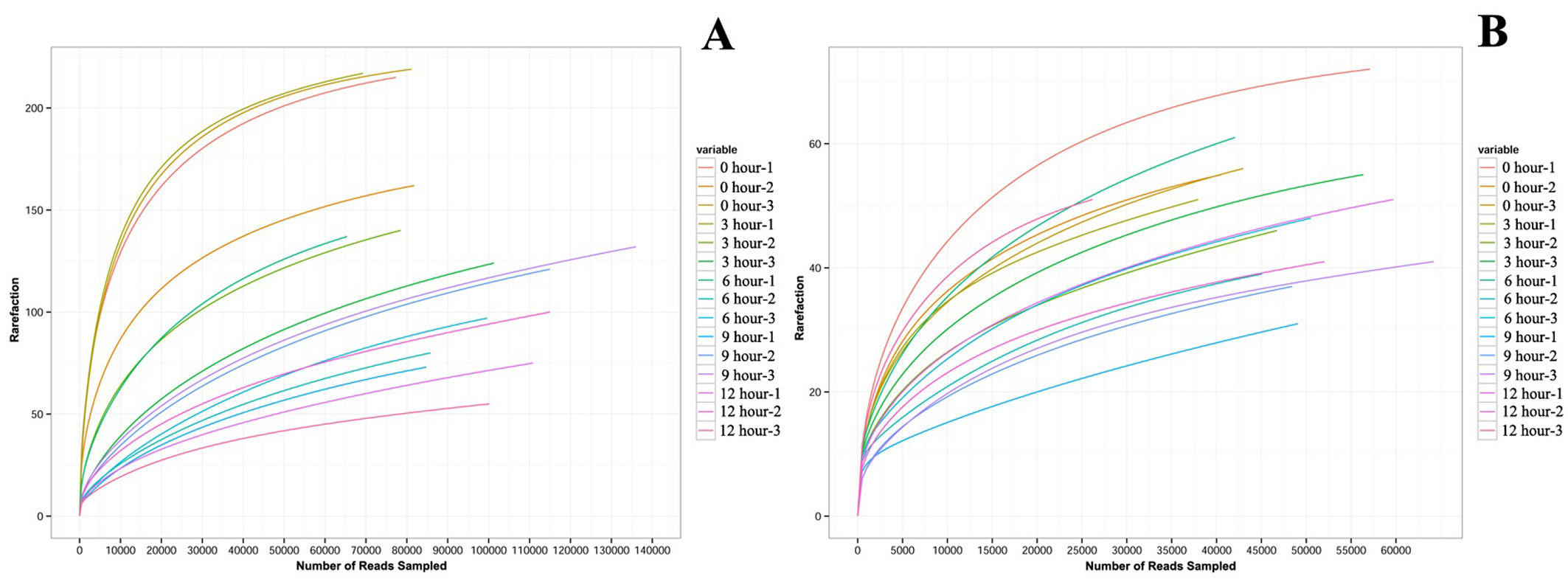

Supplement: FIGURE S1 — Rarefaction curves of fungal OTUs (A) and bacterial OTUs (B) derived from primary dark tea. [file Image_1.TIF]

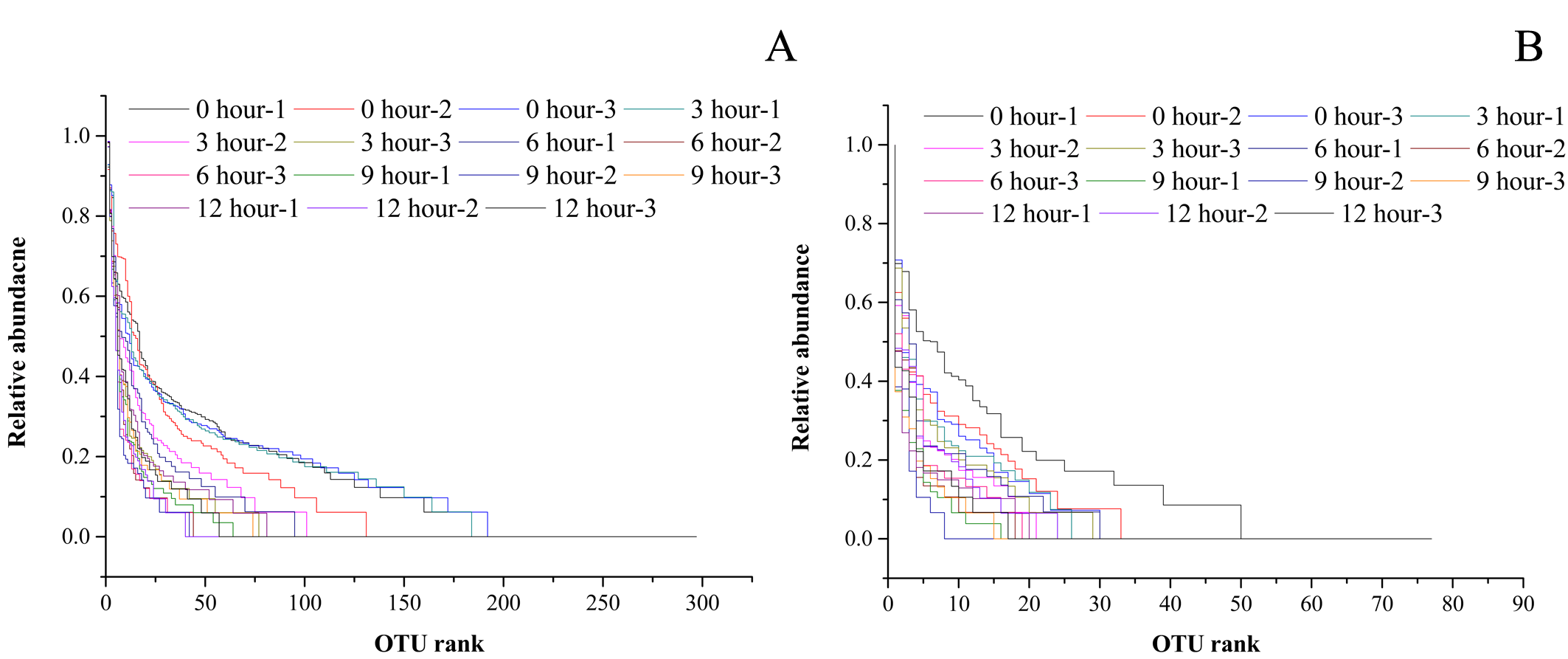

Supplement: FIGURE S2 — Rank abundance curve of fungal OTUs (A) and bacterial OTUs (B) derived from primary dark tea. [file Image_2.TIF]

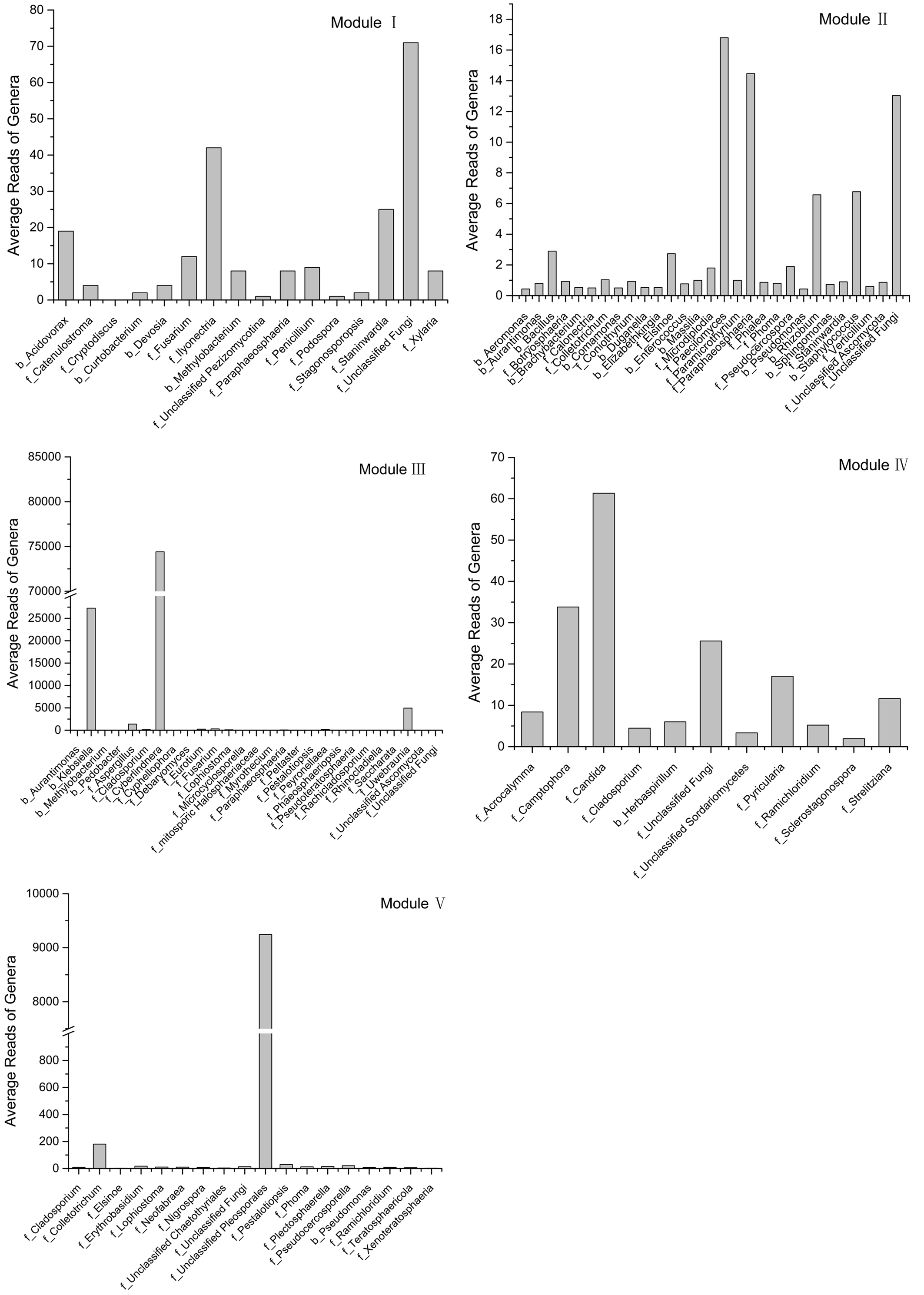

Supplement: FIGURE S3 — Microbial community composition of each co-occurrence network module. [file Image_3.TIF]

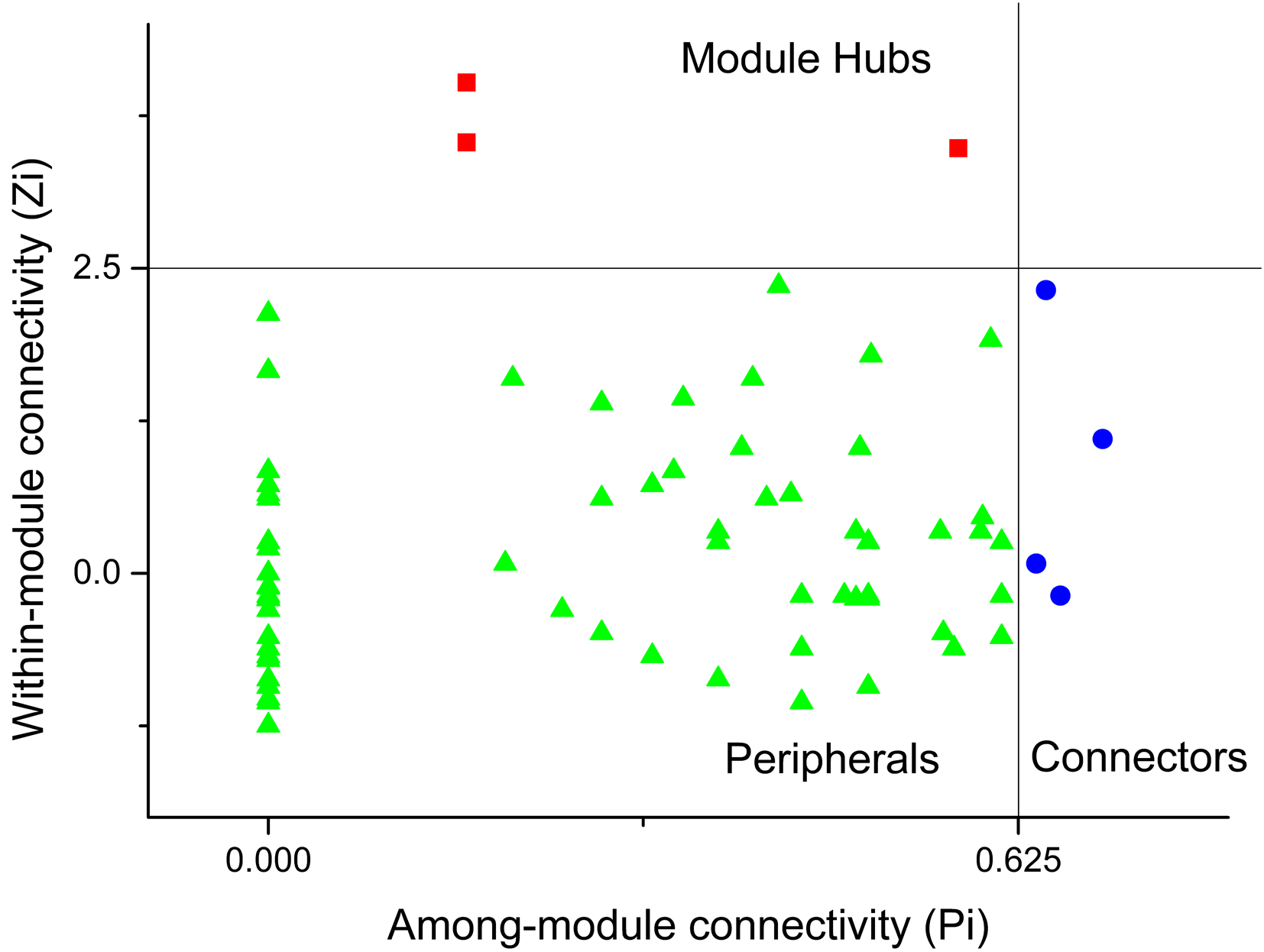

Supplement: FIGURE S4 — ZP plot showing distribution of OTUs based on their module-based topological roles. [file Image_4.TIF]
