# Supplementary material for: Biochemical Components Associated With Microbial Community Shift During the Pile-Fermentation of Primary Dark Tea
Source: Front Microbiol. 2018 Jul 10;9:1509. doi: 10.3389/fmicb.2018.01509 (PMC6048958; doi:10.3389/fmicb.2018.01509)
Supplement: TABLE S1 — Comparison of microbial diversity estimation and coverage at 97% similarity from the sequencing analysis. [file Table_1.docx]

Supplementary Material

**Table S1 Comparison of microbial diversity estimation and coverage at 97% similarity from the sequencing analysis**

| Bacteria | | | |  | Fungus | | |
| --- | --- | --- | --- | --- | --- | --- | --- |
| Sample | **No. of reads** | **No. of OTUs** | **Coverage** |  | **No. of reads** | **No. of OTUs** | **Coverage** |
| 0 hour-1 | 3223 | 60 | 0.997382 |  | 77287 | 210 | 0.99863 |
| 0 hour-2 | 8905 | 44 | 0.998473 |  | 81810 | 161 | 0.99443 |
| 0 hour-3 | 13938 | 44 | 0.995463 |  | 81091 | 213 | 0.99647 |
| 3 hour-1 | 10867 | 36 | 0.999352 |  | 69281 | 210 | 0.99645 |
| 3 hour-2 | 30238 | 33 | 0.999732 |  | 78451 | 137 | 0.99547 |
| 3 hour-3 | 32420 | 43 | 0.998263 |  | 101300 | 124 | 0.99832 |
| 6 hour-1 | 26052 | 46 | 0.997483 |  | 65357 | 134 | 0.99463 |
| 6 hour-2 | 30823 | 27 | 0.997954 |  | 85796 | 79 | 0.99846 |
| 6 hour-3 | 34615 | 34 | 0.999468 |  | 99644 | 96 | 0.99764 |
| 9 hour-1 | 35842 | 36 | 0.998943 |  | 99866 | 139 | 0.99748 |
| 9 hour-2 | 34124 | 21 | 0.994854 |  | 84750 | 73 | 0.99653 |
| 9 hour-3 | 37560 | 28 | 0.999754 |  | 114982 | 119 | 0.99843 |
| 12 hour-1 | 46064 | 30 | 0.999634 |  | 136003 | 130 | 0.99926 |
| 12 hour-2 | 41669 | 38 | 0.998574 |  | 110831 | 75 | 0.99893 |
| 12 hour-3 | 32549 | 29 | 0.995748 |  | 114998 | 98 | 0.99749 |
