# Supplementary material for: Biochemical Components Associated With Microbial Community Shift During the Pile-Fermentation of Primary Dark Tea
Source: Front Microbiol. 2018 Jul 10;9:1509. doi: 10.3389/fmicb.2018.01509 (PMC6048958; doi:10.3389/fmicb.2018.01509)
Supplement: TABLE S2 — Topological properties of network during the pile-fermentation process of primary dark tea. [file Table_2.docx]

Supplementary Material

**Table S2 Topological properties of network during the pile-fermentation process of primary dark tea**

| Network Indexes | MR01(0.790) |
| --- | --- |
| Total nodes | 124 |
| Total links | 339 |
| R square of power-law | 0.801 |
| Average degree (avgK) | 5.468 |
| Average clustering coefficient (avgCC) | 0.222 |
| Average path distance (GD) | 3.661 |
| Geodesic efficiency (E) | 0.333 |
| Harmonic geodesic distance (HD) | 3.003 |
| Maximal degree | 27 |
| Nodes with max degree | fOTU156 |
| Centralization of degree (CD) | 0.178 |
| Maximal betweenness | 1673.46 |
| Nodes with max betweenness | fOTU162 |
| Centralization of betweenness (CB) | 0.204 |
| Maximal stress centrality | 12659 |
| Nodes with max stress centrality | fOTU162 |
| Centralization of stress centrality (CS) | 1.56 |
| Maximal eigenvector centrality | 0.309 |
| Nodes with max eigenvector centrality | fOTU19 |
| Centralization of eigenvector centrality (CE) | 0.261 |
| Density (D) | 0.044 |
| Reciprocity | 1 |
| Transitivity (Trans) | 0.313 |
| Connectedness (Con) | 0.952 |
| Efficiency | 0.961 |
| Hierarchy | 0 |
| Lubness | 1 |
