# Supplementary material for: Biochemical Components Associated With Microbial Community Shift During the Pile-Fermentation of Primary Dark Tea
Source: Front Microbiol. 2018 Jul 10;9:1509. doi: 10.3389/fmicb.2018.01509 (PMC6048958; doi:10.3389/fmicb.2018.01509)
Supplement: Supplementary file 8 [file Presentation_1.pdf]

Supplementary Material

Supplementary material 1

Enzyme analysis

For PPO activity analysis, the reaction mixture consisted of 1 mL enzyme solution, 2 mL citrate buffer pH5.6, 0.4 mL 0.1% proline and 0.6 mL 1% catechol, and was incubated at 37℃° for 10 min. After the reaction was stopped by adding 1 mL 1 mol/L trichloroacetic acid and centrifuged at 15000g×g for 10 min at 4℃°, the enzyme activity was measured at 460 nm against a blank solution. One unit (U) of PPO activity was defined as the amount of enzyme catalyzing increase of 0.1 absorbance per min of 1g sample.

For POD activity analysis, the reaction mixture consisted of 0.5 mL enzyme solution, 1.5 mL 0.05 M acetic acid buffer pH 4.75, 1 mL 0.3% guaiacol and 1 mL 0.3% hydrogen peroxide, and was incubated at 35℃° for 5min. The enzyme activity was measured at 470 nm against a blank solution. One unit (U) of POD activity was defined as the amount of enzyme catalyzing increase of 0.1 absorbance per min of 1g sample.

For CEL activity analysis, the reaction mixture consisted of 1 mL enzyme solution, 4 mL sodium carboxymethylcellulose solution and incubated at 50℃° for 30min. Then added 1 mL 2M NaOH and 2.5 mL dinitrosalicylic acid (DNS) solution and the enzyme activity was measured at 520nm against glucose standards curve. One unit (U) of CEL activity was defined as the amounts of enzyme produced 1 mg glucose per min of 1g sample.

For PEC activity analysis, the reaction mixture consisted of 0.5mL enzyme solution, 1mL acetic acid buffer pH 4.8, 0.5mL pectin solution and was incubated at 48℃° for 30min. Then placed in boiled water for 5min and added 2.5mL DNS solution. The PEC activity was measured at 540nm against galacturonic acid standards curve. One unit (U) of PEC activity was defined as the amounts of enzyme produced 1 mg galacturonic acid per min of 1g sample.
